# Microfluidic-Chip-Based Formulation and In Vivo Evaluations of Squalene Oil Emulsion Adjuvants for Subunit Vaccines

**DOI:** 10.3390/vaccines12121343

**Published:** 2024-11-28

**Authors:** Shashank Bhangde, Stephanie Fresnay-Murray, Tyler Garretson, Asma Ashraf, Derek T. O’Hagan, Mansoor M. Amiji, Rushit N. Lodaya

**Affiliations:** 1 Department of Pharmaceutical Sciences, School of Pharmacy and Pharmaceutical Sciences, Bouvé College of Health Sciences, Northeastern University, Boston, MA 02115, USAm.amiji@northeastern.edu (M.M.A.); 2 GSK, Rockville Centre for Vaccines Research, Rockville, MD 20850, USA; 3 Department of Chemical Engineering, College of Engineering, Northeastern University, Boston, MA 02115, USA

**Keywords:** adjuvants, microfluidic chip, nanoemulsions, process optimization, subunit vaccines, microfluidics

## Abstract

Background: Adjuvants play a crucial role in improving the immunogenicity of various antigens in vaccines. Squalene-in-water emulsions are clinically established vaccine adjuvants that improve immune responses, particularly during a pandemic. Current manufacturing processes for these emulsion adjuvants include microfluidizers and homogenizers and these processes have been used to produce emulsion adjuvants to meet global demands during a pandemic. These processes, however, are complex and expensive and may not meet the global needs based on the growing populations in low- and middle-income countries. At the forefront of adjuvant research, there is a pressing need to manufacture emulsion adjuvants using novel approaches that balance efficacy, scalability, speed of production, and cost-effectiveness. Methods: In this study, we explored the feasibility of a microfluidic chip platform to address these challenges and evaluated the adjuvanticity of the emulsion adjuvant prepared using the microfluidic chip process in CB6F1 mice model, and compared it with a control formulation. We developed and optimized the process parameters to produce emulsion adjuvants with characteristics similar to SEA160 (control formulation). Results: The resulting emulsion prepared using the microfluidic chip process (MC160) when mixed with ovalbumin, maintained antigen structural integrity. Immunogenicity studies in a CB6F1 mouse model, with the Cytomegalovirus glycoprotein B (CMV gB) antigen, resulted in humoral responses that were non-inferior between MC160 and SEA160, thereby validating the microfluidic chip approach for manufacturing emulsion adjuvants. Conclusions: These findings demonstrate a proof of concept for using microfluidic chip platforms for formulating emulsion adjuvants, offering a simpler manufacturing platform that can be deployed to low- and middle-income countries for rapid production, improving adjuvant access and aiding in pandemic preparedness.

## 1. Introduction

Vaccine adjuvants are critical for enhancing the immunogenicity of antigens, which in turn improve vaccine efficacy [1]. Squalene, a biodegradable triterpene oil abundantly found in the shark liver, is a component of clinically used oil-in-water (O/W) emulsion adjuvants such as MF59 and AS03 [2,3,4,5,6]. Squalene-based emulsion adjuvants play a key role in vaccine adjuvants by improving both innate as well as adaptive immune responses and have demonstrated improved cross-protectivity, particularly for seasonal and pandemic influenza vaccines [6,7,8,9,10,11]. These emulsion adjuvants help with both reducing the antigen dose as well as reducing the number of immunizations necessary, thereby making them a suitable choice for inclusion in various subunit vaccines including the COVID-19 vaccines [11,12,13,14,15]. Current manufacturing processes for squalene oil emulsions include equipment such as microfluidizers and homogenizers [3,16,17]. Although these processes can manufacture emulsion adjuvants on a larger scale rapidly to meet increasing demands, they are capital-intensive in terms of both housing as well as maintenance costs. These processes also require both a complex manufacturing infrastructure as well as equipment maintenance [3,18]. The rapid deployment of these adjuvants to low- and middle-income countries (LMICs) to improve vaccine access demands a cost-efficient, as well as simpler, process for manufacturing these emulsions [8,11,19,20]. Scalability for industrial production for promising simpler methods like self-emulsification remains a current challenge [21,22]. In the rapidly developing field of adjuvant research, there is a need for innovative approaches for manufacturing emulsion adjuvants that can balance vaccine immunogenicity, scalability, production speed, and cost-effectiveness [6,23].

Microfluidics, a technology involving fluid manipulation at micrometer dimensions, has shown application for the rapidly scalable production of nanoparticles, as evident from the recent COVID-19 vaccine campaigns [24,25,26,27]. Microfluidic devices allow for precise control over fluid dynamics at the microscale, which will enable the production of highly uniform and reproducible emulsions [24,28]. This technology has the potential to streamline the manufacturing process by reducing complexity and the need for large spaces for bulky instruments and increasing accessibility [29]. Both the affordability aspects as well as the simplicity of the process of the microfluidic chip platform make it suitable to deploy the technology for simpler and rapid production, which would improve adjuvant access and aid in pandemic preparedness in LMICs [27,30]. We hypothesize the use of microfluidic-chip-based technology as an alternative approach to manufacturing reproducible squalene oil emulsions similar in characteristics to previously established emulsion adjuvants.

In this manuscript, we investigate the feasibility of using a microfluidic chip platform to produce squalene oil emulsions for use as vaccine adjuvants. Utilizing the Precision Nanosystems NanoAssemblr^®^ Ignite™ and NxGen™ (Vancouver, BC, USA) microfluidic chip, we developed and optimized the emulsification process to create adjuvants with characteristics comparable to the control formulation, self-emulsifying adjuvant 160 (SEA160) [21,31]. The microfluidic-chip-produced emulsion (MC160) was subjected to accelerated stability for up to four weeks to evaluate the size and polydispersity index (PDI), and also antigen structural integrity, when combined with ovalbumin (OVA), a model antigen. In vivo immunization studies were conducted in female CB6F1 mice using a poorly immunogenic CMV gB antigen to evaluate humoral immune responses elicited by MC160 compared to SEA160. Additionally, antibody subclasses of IgG were investigated from harvested sera to test the presence of antigen-specific IgG1 antibody (Th2-type) immune responses that are typical to emulsion adjuvants [32]. Overall, the work in this manuscript will provide a proof of concept for manufacturing emulsion adjuvants using a microfluidic-chip-based platform that, on integration, could help address and improve global vaccine access for adjuvants and aid in pandemic preparedness.

## 2. Materials and Methods

### 2.1. Formulation Materials

Squalene, span-85, and polysorbate-80 (PS-80) were procured from MP Biomedicals (Irvine, CA, USA), Sigma-Aldrich (St. Louis, MO, USA), and Acros Organics (Geel, Belgium), respectively. Ethanol and isopropyl alcohol (IPA) were procured from VWR Chemicals (Radnor, PA, USA). Sodium citrate buffer stock (100 mM, pH 6.0) and 1× PBS were procured from Teknova (Hollister, CA, USA) and Corning (Manassas, VA, USA), respectively. Cell-line derived CMV gB used to evaluate the in vivo immunogenicity of the novel adjuvants in mice was provided by GSK (Rockville, MD, USA).

### 2.2. Process Development and Optimization

To make emulsion adjuvants using the microfluidic chip platform, NanoAssemblr^®^ Ignite™ and NxGen™ microfluidic chips from Precision Nanosystems (Vancouver, BC, USA) were used. BD syringes (Franklin Lakes, NJ, USA) (1 mL, 3 mL, 5 mL, 10 mL) were found to be compatible with this system, and volume-appropriate syringes were used. The composition of the emulsions prepared using the microfluidic chip process was set to be the same as that of SEA160 (final concentrations of 3.5% squalene, 0.75% span-85, 0.75% PS-80 in 10 mM citrate buffer, pH 6.5) [21,31]. The process includes aqueous (AP) and organic (OP) phases. The two phases were fed into the chip at the set flow rate (in mL/min) and the set flow ratio (OP:AP). Start and end waste were set to 0.30 mL and 0.10 mL, respectively. The resultant formulation from the chip was a milky-white nanoemulsion (in ethanol). Particle size and polydispersity index (PDI) were measured after the formulation step as an in-process quality control step. One-parameter-at-a-time approach was used to optimize the parameters.

**Figure 1:**
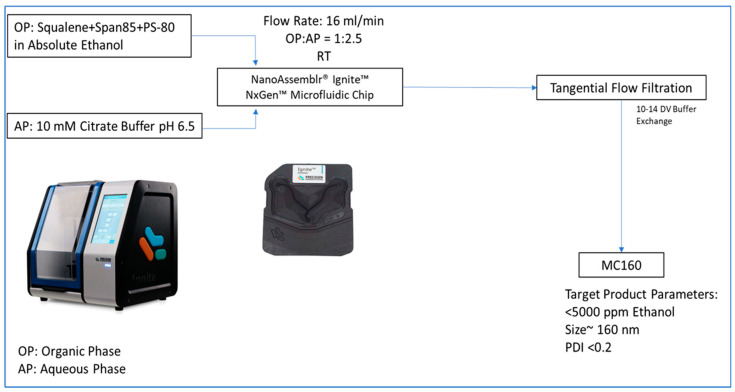
Optimized microfluidic chip process of formulating squalene oil emulsions. Process description: The process includes two phases, aqueous (AP) and organic (OP). The two phases were fed into the NxGen™ microfluidic chip on Precision Nanosystems NanoAssemblr^®^ Ignite™ at the set flow rate (16 mL/min) and the set flow ratio (OP:AP = 1:2.5). The resultant formulation coming out of the chip was a milky-white nanoemulsion (in ethanol). This was then buffer-exchanged using a tangential flow filtration with 10–14 diavolumes of buffer exchange, yielding MC160 with our desired target product parameters.

Phase composition was the first parameter to be optimized. The phase compositions that were evaluated were as follows—OP(1): squalene and span-85 in ethanol, AP(1): PS-80 in 10 mM citrate buffer and OP(2): squalene, span-85, and PS-80 in ethanol, AP(2): 10 mM citrate buffer. The emulsions were formulated at 10 mL/min and a flow ratio of 1:1. Following phase composition, the flow ratios were optimized where the starting point of OP:AP ratio was 1:1. The final concentration of the emulsion was set to 3.5% squalene, 0.75% span-85, and 0.75% PS-80 (similar to SEA160) irrespective of the flow ratio [21,31]. The flow rate was set to 14 mL/min and the mixing ratios from 1:1, 1:1.5, 1:2, 1:2.5, 1:3, 1:4, and 1:5. The flow rates were optimized at the flow ratio of 1:2.5 (OP:AP). The flow rates assessed were 6–20 mL/min. Figure 1 shows the optimized microfluidic chip process of formulating squalene oil emulsion adjuvant.

### 2.3. Tangential Flow Filtration

Pellicon^®^ XL ultrafiltration 10 kDa cassette (Darmstadt, Germany) was used to perform buffer exchange. Polysulfone pressure transducers were procured from Repligen (Waltham, MA, USA) and used to measure feed, permeate, and retentate pressures of filtration assembly. Tangential flow filtration assembly items including feed pump, buffer inlet pump, and balances were procured from Spectrum Labs (Los Angeles, CA, USA). To replace ethanol from the product after formulation with the microfluidic chip, tangential flow filtration was used. The product (from the microfluidic chip process) was added to the feed container and fed into the TFF cassette with the help of a pump. The retentate line was fed back into the feed tank. The filtrate (containing buffer and ethanol) was collected and weighed. The diafiltration buffer tank contained the background buffer (10 mM sodium citrate buffer, pH 6.5). The input flow rate of the buffer from the diafiltration buffer tank to the feed tank was monitored and was set equivalent to the filtrate output rate. A residual ethanol study was performed to determine the amount of ethanol in the feed tank after every two diavolumes of buffer exchange. The residual ethanol in the product was determined using a gas chromatography–flame ionization detector (GC-FID) from Thermo Scientific (Waltham, MA, USA) [33].

### 2.4. Formulation Characterization

Particle sizes and polydispersity index (PDI) were measured using dynamic light scattering (DLS) from the Malvern Zetasizer Nano ZS (Malvern, UK) (100× dilution in DI water). Osmolality was measured using freezing point depression on an Advanced Instruments model 2020 osmometer (Norwood, MA, USA). pH was measured using the Orion VersaStar pH meter from Thermo Scientific (Waltham, MA, USA) and was calibrated using a three-point curve. Endotoxin levels were measured using Endosafe^®^ nexgen-PTS™ cartridges (0.1–10 EU/mL) and the Endosafe^®^ nexgen-MCS™ instrument from Charles River laboratories (Wilmington, MA, USA) using manufacturer recommendations (10× diluted samples in provided endotoxin-specific buffer). Squalene content was determined using reverse-phase–high-performance liquid chromatography (RP-HPLC) [31] and ethanol content in formulations was determined using GC-FID [33].

### 2.5. Antigen Integrity

#### 2.5.1. Gel Electrophoresis

Antigen integrity was determined using sodium-dodecyl-sulphate-polyacrylamide gel electrophoresis (SDS-PAGE). Ovalbumin was procured from InvivoGen (San Diego, CA, USA). Beckman Coulter Airfuge Air-Driven Ultracentrifuge (Indianapolis, IN, USA) was used to disrupt the emulsion droplets. Gel running assembly was procured from Invitrogen (Carlsbad, CA, USA), MES SDS Buffer (20×) was procured from Thermo Scientific (Waltham, MA, USA), and PowerPac HC power supply was procured from Bio-Rad (Hercules, CA, USA). NuPAGE 4–12% Bis-Tris Gel and NuPAGE LDS Sample Buffer (4×) were procured from Invitrogen. PageRuler Plus Prestained Protein Ladder was used as a marker and Imperial Protein Stain was used to stain the gels and these were procured from Thermo Scientific (Waltham, MA, USA). Deionized (DI) water was used for destaining the gels. To determine the stability of the antigen when mixed with the adjuvant and stored at 4 °C, an in vitro integrity test using ovalbumin (OVA) as the model antigen for up to 48 h post mixing was performed. OVA was mixed with MC160 in a 1:1 ratio to mimic mixing before administration in an adjuvanted vaccine. OVA in 1× PBS (40 μg/mL) was prepared and mixed with the adjuvants and stored for 0, 2, 5, 24, and 48 h at 4 °C. The formulations were mixed in a reverse pattern such that samples from all the timepoints were run on the same gel. At the end of the timepoint, emulsion droplets were disrupted by air-driven ultracentrifugation (25 psi, 20 min). The subnatant was collected and mixed with 4× sample buffer containing dithiothreitol (DTT) and bromophenol blue. These samples were heated at 95 °C for 10 min and snap-cooled on ice. All samples were loaded on a 4–12% bis-tris gradient gel with a protein ladder. Stand-alone OVA and adjuvant samples were also run on the same gel as positive and negative controls, respectively. The samples were run using 1× MES SDS running buffer at 200 V (constant voltage mode) for 35–45 min. After the run, the gel was carefully transferred to a staining tray and washed thrice with 50 mL of DI water (5 min each wash on a shaker) and then placed into 25 mL of protein stain for two hours at RT on a shaker. After two hours, the gel was washed thrice with 50 mL of DI water (5 min each wash on a shaker) to remove excess stain. The gel was then destained overnight in DI water to remove non-specific staining and was scanned on a Gel-doc imaging system from Bio-Rad and analyzed using the Image Lab image processing software (version 3.0).

#### 2.5.2. Circular Dichroism

Antigen integrity was determined using circular dichroism (CD). Circular dichroism J-1500 Spectrometer and Spectra™ manager Software from Jasco (Easton, MD, USA) (version 2) were used. One mm pathlength quartz cuvette with stopper was procured from Jasco (Easton, MD, USA). To determine the structural stability of the antigen, when mixed with the adjuvant and stored at 4 °C, far-ultraviolet CD (FUV CD) measurements were conducted using ovalbumin (OVA) as the model antigen for up to 48 h post mixing. OVA in 1× PBS (37.5 mg/mL) was prepared and mixed in a 1:1 volume ratio with the emulsion adjuvants (SEA160 and MC160) and stored for 0, 2, 6, 24, and 48 h and at 4 °C. The formulations were mixed in a reverse pattern where all timepoints would end in a closer time. In the end, all samples were diluted 100-fold in background buffer (1:1 1× PBS: 10 mM citrate buffer, pH 6.5) and loaded onto a quartz cuvette and FUV CD measurements were recorded. For each sample, two measurements of three cycles were recorded. Additionally, OVA, adjuvant, and background buffer were measured at the same dilutions as samples. Captured CD data were buffer-subtracted, offset-corrected (250–260 nm), and converted to specific ellipticity with the help of the equation below [34]. The molar ellipticity data for each measurement were obtained at 0.5 nm intervals (200–260 nm).

$$Molar\;Ellipticity\;\theta_{\lambda }^{exp}=\frac{\theta_{\lambda }^{raw}}{1000}\times \frac{M}{C_{soln}\times L}$$

In the above equation, molar ellipticities $\left(\theta_{\lambda }^{exp}\right)$ are in deg×cm^2^/dmol, M is the mean residue molecular weight of 112 g/mol, $\theta_{\lambda }^{raw}$ is background corrected raw CD signal in deg, L is the path length of the cuvette in mm, and $C_{soln}$ is the solution concentration of the protein in mg/mL. Molar ellipticities as a function of wavelength were plotted for all the timepoints of each sample set. The molar ellipticities for each sample were compared with other timepoints and normalized root mean square deviation (% NRMSD) values were calculated for duplicates of samples and references using an R script. Percent NRMSD values were calculated based on the equation below [35]. Percent NRMSD values of less than 10% for references and samples indicated that the references and samples were in close agreement [35,36,37].

$$\%NRMSD\;=\frac{\sqrt{\frac{\sum_{\lambda =200}^{260}{\left(\theta_{\lambda ,Reference}^{exp}-\theta_{\lambda ,Sample}^{exp}\right)}^{2}}{121}}}{\theta_{Reference,max}^{exp}-\theta_{Reference,min}^{exp}}\times 100$$

#### 2.5.3. Differential Scanning Fluorimetry

Differential scanning fluorimetry (DSF) was used to determine the thermal unfolding temperature (Tm) of the protein. Prometheus NT.48 NanoDSF instrument and high-sensitivity capillaries were procured from NanoTemper Technologies (San Francisco, CA, USA). ThermControl Software (Munich, Germany) (version 2.3.1) was used to capture and analyze data. To determine the structural stability of the antigen when mixed with the adjuvant and storage at 4 °C, Tm was determined. OVA in 1× PBS (37.5 mg/mL) was prepared and mixed in a 1:1 volume ratio with the emulsion adjuvants (SEA160 and MC160) and stored for 0, 2, 6, 24, and 48 h and at 4 °C. The formulations were mixed in a reverse pattern where all time points would end in a closer time. At the end, samples were loaded on the capillaries (two capillaries for each sample), and raw data were captured. The raw data generated were the ratio of fluorescence intensity (330 nm/350 nm) as a function of temperature. The first derivative of the data was plotted to determine the transition temperatures of unfolding as maxima as a function of the temperature. The software provided Tm (mid-point) of thermal unfolding from the first derivate of the Fl spectra. The first derivative also provided the onset temperature at which the fluorescence intensity ratio between 330 nm and 350 nm began to shift indicative of the initiation of unfolding. The ratio of fluorescence intensity (330 nm/350 nm) and first derivatives were plotted. Tm values, presented as the mean of duplicates, were plotted for all samples, and compared to OVA.

### 2.6. In Vivo Immunogenicity Evaluation of CMV gB Antigen with Emulsion Adjuvants

#### 2.6.1. Ethics Statement

All studies were conducted in accordance with the GSK requirements and process on the Care, Welfare, and Treatment of Laboratory Animals and were reviewed by the Institutional Animal Care and Use Committee (IACUC; Protocol No: AUP0923) through the ethical review process at the institution where the work was performed [38]. All studies were executed in compliance with provisions from the USDA Animal Welfare Act (AWA), the Public Health Service Policy on Humane Care and Use of Laboratory Animals, the US Interagency Collaborative Animal Education (ICARE) Project, and the Committee Principles for the Utilization and Care of Research Animals [39]. The study was conducted in partnership with GSK Upper Providence, PA.

#### 2.6.2. Study Design and Immunization Regime

(CMV gB) was used as a model antigen to evaluate the immunogenicity of emulsion adjuvants in mice [40]. Five- to eight-week-old female CB6F1 mice (Charles River Laboratories, Rayleigh, NC, USA) were immunized three times at three-week intervals with 50 µL of either saline, unadjuvanted CMV gB, CMV gB + SEA160, or CMV gB + MC160. CMV gB antigen dose for each immunization was set to 0.25 µg. Each mouse received 50 µL of the vaccine injected intramuscularly into the quadriceps such that at each timepoint an alternate hind limb was used. The vaccine was formulated to mimic bedside immunization, with a 1:1 mixing of antigen and adjuvant. A sample size of ten animals per group was calculated to provide a power of 80% to detect at least a three-fold difference between any two groups with a 95% confidence interval. Blood was collected 3 weeks post-first (3wp1), 3 weeks post-second (3wp2), and 3 weeks post-third (3wp3) immunizations and sera were isolated to assess the humoral immune responses by determining the neutralizing antibody and binding antibody titers (CMV gB-specific total IgG and subclass IgG titers).

#### 2.6.3. Neutralizing Antibody Assay

Phosphate-buffered saline (1× PBS) used throughout the assay was 1× sterile PBS (w/o Ca^2+^ and Mg^2+^, pH 7.4) unless stated otherwise. All other reagents used for this assay were sterile and of cell-culture grade. The retinal pigment epithelial cell line (ARPE-19) was procured from ATCC (Manassas, VA, USA). TB40 (a CMV strain) virus stocks were grown in-house in ARPE-19 cells from previous stocks [41]. The working medium (R0) used for this assay was composed of Dulbecco’s Modified Eagle Medium (DMEM) (with L-glutamine) + 10% (*v*/*v*) fetal bovine serum (FBS) + 1% (*v*/*v*) penicillin-streptomycin (PS). Lyophilized guinea pig complement was procured from Cederlane (Burlington, NC, USA) as it helps in reducing background. Each vial of lyophilized complement was solubilized in 50 mL of working media (R0) and filtered through a 0.2 µm filtration unit to yield 50 mL of prepared media with guinea pig complement (R1). These prepared media with guinea pig complement (R1) were used to prepare the TB40 virus for day 2. A positive control (PC) known to neutralize the TB40 virus was procured from Sera Care (Milford, MA, USA). Mouse anti-CMV IE1 monoclonal antibody from EMD Millipore (Burlington, MA, USA) was used as the primary antibody. Goat anti-mouse IgG Alexa Fluor 488 from Invitrogen (Carlsbad, CA, USA) and DAPI from Thermo Scientific (Waltham, MA, USA) were used as secondary antibodies. Gelatin Type B was procured from Fisher Chemicals (Waltham, MA, USA). Gelatin solution was prepared in 1× PBS (concentration: 0.2% (*w*/*v*)) and used for making antibody dilutions (PBS-GB). Tecan Fluent^®^ Automation Workstation (liquid handling robot) and BioTek EL406 plate Washer Dispenser were used for the assay. High-content imaging was conducted using the Thermo Scientific Cellinsight CX7 LED Pro High-Content Screening Platform connected to a Thermo Scientific Orbitor RS Microplate Mover (Waltham, MA, USA). ARPE-19 cells were used for this assay since TB40 is known to infect these cells [18,42]. On day one, 100 µL of ARPE-19 cells (8.5 × 10^4^ live cells/mL, average viability > 95%) were plated in 96-well flat- and clear-bottom plates in working medium (R0). Plates (with lids) were incubated in humidified cell culture incubators at 37 °C, 5% CO_2_ (95% air) overnight (~24 h). On Day 2, the Tecan Fluent^®^ Automation Workstation was used to perform serum dilutions. Different starting dilutions were used for different timepoints depending on the expected titers. A positive control was used on every plate at a constant 1:50 starting dilution. In each plate, a 75 µL serum dilution was prepared using Tecan Fluent^®^ Automation Workstation, and then 75 µL of TB40 virus (4 µL TB40 virus + 71 µL prepared media with guinea pig complement (R1)) was added to each well to make a total of 150 µL in each plate. This virus–serum mixture was incubated in humidified cell culture incubators at 37 °C, 5% CO_2_ (95% air) for 2 h. The cell plates (duplicate for each group) were removed from the incubator, working media were taken out from each well, and 50 µL of incubated virus–serum mixture was added to the cell plates. These cell plates were incubated in humidified cell culture incubators at 37 °C, 5% CO_2_ (95% air) for at least 20 h. On Day 3, the virus–serum mixture was removed from the plates, and the plates were washed with 1× PBS (50 µL/well) and then fixed using 4% paraformaldehyde (in 1× PBS) (50 µL/well) and incubated at RT for 20 min followed by one wash using 1× PBS (50 µL/well). The cell monolayer was permeabilized using 0.1% TritonX-100 (in 1× PBS) (50 µL/well) and then incubated for another 10 min at RT. After two washes with 1× PBS (50 µL/well), primary antibody (1:1000 dilution in PBS-GB) (50 µL/well) was added immediately and the plates were incubated for 1 h in humidified cell culture incubators at 37 °C, 5% CO_2_ (95% air). Cells were washed twice with 1× PBS (50 µL/well) and then a secondary antibody (goat anti-mouse IgG AlexaFlour488 and DAPI) (1:1000 and 1:2000 dilution in PBS-GB, respectively) (50 µL/well) was added, and the plates were incubated for another 1 h in humidified cell culture incubators at 37 °C, 5% CO_2_ (95% air). Post incubation, the plates were washed thrice with 1× PBS (50 µL/well), and 1× PBS (100 µL/well) was added. These plates were sealed with transparent adhesive film (Thermo Fisher Scientific MicroAmp™ Optical Adhesive Film (Waltham, MA, USA)) and were then read using high-content imaging by the CX7 system (by selecting to read 10–20 fields per well). Interpolated titers were then calculated at 50% fluorescence intensity. GraphPad Prism software (San Diego, CA, USA) (version 9) was used to analyze and plot data for the neutralizing antibody titers. Statistical analyses were performed on titers post-log transformation using one-way ANOVA followed by Tukey’s test to compare groups with each other [43].

#### 2.6.4. Binding Antibody Assay

A high-throughput assay to determine CMV-gB-specific binding antibody titers (Total and subclass IgG) was developed using the Luminex™ FLEXMAP 3D™ Instrument System from Life Technologies (Austin, TX, USA). The magnetic beads utilized for our assays were Bio-Plex Pro Magnetic COOH Beads procured from Bio-Rad (Hercules, CA, USA). COOH-bound magnetic beads were used to couple the CMV gB antigen [44]. R-Phycoerythrin (rPE) conjugated goat anti-mouse IgG detection antibodies (total IgG and IgG subclasses (IgG1, IgG2a, IgG2b)) were procured from Jackson Immunoresearch Labs (West Grove, PA, USA). Phosphate-buffered saline (1× PBS) used throughout the assay was 1× sterile PBS (w/o Ca^2+^ and Mg^2+^, pH 7.4) unless stated otherwise. All other reagents used for this assay were of laboratory grade. The assay buffer used for this experiment was 1% (*w*/*v*) bovine serum albumin (BSA) and0.05% (*v*/*v*) sodium azide in 1× PBS and the wash buffer used for this experiment was 0.05% Tween-20 in 1× PBS. Agilent (Santa Clara, CA, USA) BioTek 405 microplate washer with magnetic insert (to hold the magnetic beads in place) was used for the assay. SoftMax Pro software (Molecular Devices, San Jose, CA, USA) (version 7.3) was used to plot and analyze data. MagPlex beads were coated with a fixed amount of antigen (10 μg/mL). A positive control sample from an internal study of CMV-immunized mice was run on each plate. The serum samples were serially diluted two-fold across the plate in assay buffer starting from 1:50 dilution (final volume: 50 μL/well). The diluted sera were mixed with the antigen-coated beads (target bead concentration: 50,000 beads/mL, diluted in assay buffer, 50 μL/well), sealed with an opaque adhesive film (Black Film, Light Absorbing from USA Scientific (Ocala, FL, USA)), and incubated at room temperature for 60 min on a plate shaker. After incubation, the opaque adhesive seal was removed, and the plates were washed with a wash buffer (100 μL/well) using the plate washer (with a magnetic insert). After washing the plates carefully and thoroughly, the detection antibody solution (antibody dilution:1:50 in assay buffer) was added (50 μL/well), sealed with an opaque adhesive film, and incubated at room temperature for 60 min on a plate shaker. After incubation, the opaque adhesive seal was removed, and the plates were washed with a wash buffer (100 μL/well) using the plate washer (with a magnetic insert). After washing the plates carefully and thoroughly, 100 μL/well 1× PBS was added to each plate sealed with an opaque adhesive film and incubated at room temperature for 20 min on a plate shaker. The data were recorded and the median fluorescence intensity (MFI) for each sample was used for analysis. The plates were read on FlexMap 3D with a set protocol where 50 μL of the sample per well was drawn and 50 beads were counted. The output data (MFI) were plotted as a function of dilution factors and quantitative analysis of samples using a four-parameter logistic (4PL) curve fit was performed to determine endpoint titers (at MFI = 10,000). Using this assay, total IgG titers for three weeks post-second and third immunization serum samples and subclass IgG titers for three weeks post-third immunization serum samples *(n* = 10/group) were determined. GraphPad Prism software (San Diego, CA, USA) (version 9) was used to plot data (endpoint titers) for total and subclass IgG titers. Statistical analyses were performed on titers post-log transformation using one-way ANOVA followed by Tukey’s test to compare groups with each other [43].

## 3. Results

### 3.1. Process Definition and Optimization for Emulsion Adjuvants Using Microfluidic Chip Platform

A microfluidic chip mixer system (from Precision Nanosystems) was used for the propagation of adjuvant emulsions and involves two phases, an organic phase containing lipids and an organic solvent and an aqueous phase containing buffer. The two phases enter the two channels under laminar flow and are mixed on a microscale in a controlled fashion [45]. The rapid mixing of the two phases on a microfluidic chip allows for the formation of emulsions at the nanoscale (~100–200 nm) due to the rapid dilution of the organic phase [46]. This mixing and controlled formation of droplets is also facilitated by the geometric design of the flow path in the microfluidic chip [46,47]. NxGen™ microfluidic chips from Precision Nanosystems are equipped with the bifurcating mixer design where a controlled solvent displacement of the organic phase and aqueous phase occurs, leading to the formation of stable emulsion [45,46,48,49].

**Figure 2:**
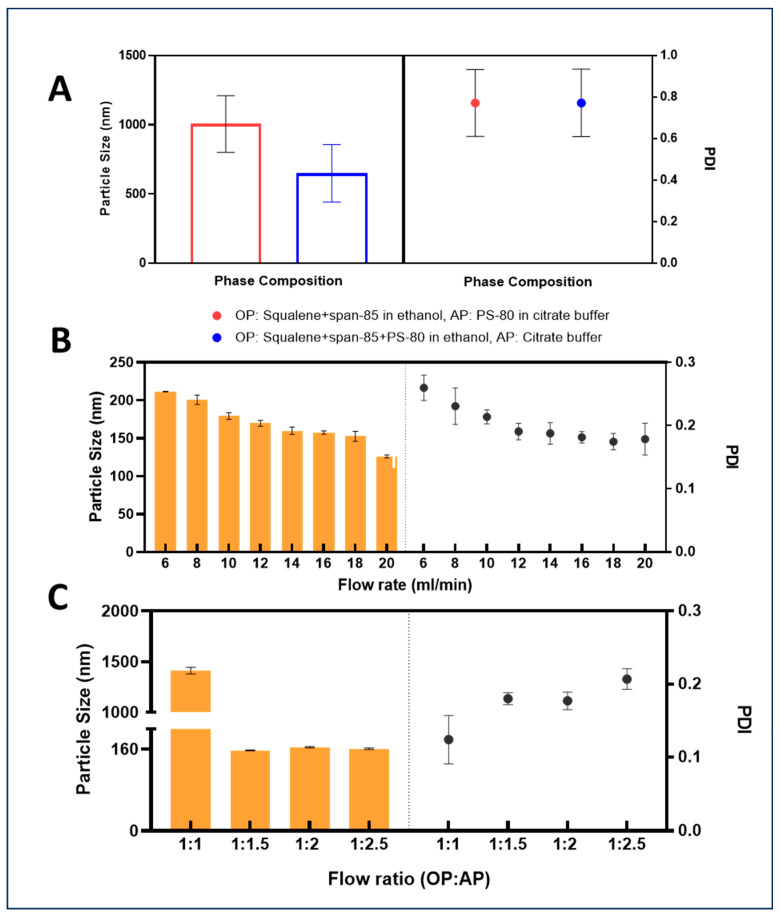
Effects of process parameters on mean droplet size (bars) and PDI (points) of squalene oil nanoemulsion prepared by microfluidic mixing. (**A**) phase composition; (**B**) flow rate; (**C**) flow ratio. Each point represents the mean value ± SD (*n* = 3).

We used the one-parameter-at-a-time approach to optimize the process parameters. While optimizing all the parameters, the desired/final concentration was set to 3.5% squalene, 0.75% span-85, and 0.75% PS-80 (similar to SEA160) [21,31]. While optimizing the phase compositions, we evaluated two combinations (Combination 1—OP(1): squalene and span-85 in ethanol, AP(1): PS-80 in 10 mM citrate buffer, pH 6.5; Combination 2—OP(2): squalene, span-85, and PS-80 in ethanol, AP(2): 10 mM citrate buffer, pH 6.5). The emulsions were formulated at 10 mL/min and a flow ratio of 1:1. It was observed that squalene and span-85 in ethanol formed particles around 1000 nm whereas the particle size for squalene, span-85, and PS-80 in ethanol was around 650 nm (Figure 2A). Both compositions showed a polydispersity index (PDI) around 0.800 (which was greater than 0.200). The droplets generated were found to be highly polydisperse and exhibited high variability in both particle size as well as PDI. Based on the smaller average particle sizes, the OP composition was optimized to squalene, span-85, and PS-80 in ethanol, and the AP composition was optimized to 10 mM sodium citrate buffer, pH 6.5. We expected that optimizing the flow ratio and flow rate would eventually reduce the particle sizes further. The flow rate was set to 14 mL/min while optimizing the flow ratio (test range: 1:1, 1:1.5, 1:2, 1:2.5, 1:3, 1:4, and 1:5). The final concentration of the emulsion was set to 3.5% squalene, 0.75% span-85, and 0.75% PS-80 (similar to SEA160) irrespective of the flow ratio [21,31]. It was observed that emulsions were not formed for flow ratios of 1:3 and above due to leaks in the process caused by the high viscosity of the organic phase (attributed to the higher concentration of squalene and surfactants in the organic phase to achieve the target concentration). For the flow ratios 1:1.5, 1:2, and 1:2.5, we found that the particle sizes and PDI were close to our target product parameters (size ~160 nm, PDI < 0.2). The flow ratio was fixed to 1:2.5 (OP:AP) since this process used the least amount of ethanol, thereby requiring lower efforts to replace ethanol in the buffer exchange process (Figure 2C). The flow ratio of 1:2.5 (OP:AP) resulted in emulsions with an approximate concentration of ethanol around 23.5% (*v*/*v*). For flow rate optimization, flow rates were evaluated from 6 to 20 mL/min at a flow ratio of 1:2.5 (OP:AP). It was observed that the flow rates of 14 and 16 mL/min resulted in emulsions with particle sizes of around 160 nm and a polydispersity index (PDI) < 0.2 (Figure 2B). The flow rate was set to 16 mL/min.

After optimization of the microfluidic chip process parameters, tangential flow filtration was evaluated as the process to buffer-exchange the formulation to eliminate ethanol. A residual ethanol study was performed to determine the amount of ethanol in the feed tank after every two diavolumes of buffer exchange. The residual ethanol in the product was determined using gas chromatography–flame ionization detection (GC-FID) [33]. We also monitored the size and PDI throughout the process and it was observed that the buffer exchanged did not have an impact on the particle size and PDI of the formulation. The residual ethanol in the formulations was found to be below 2000 ppm after 6–8 diavolumes of buffer exchange (Figure S1). Thus, the number of diavolumes of buffer exchange to be performed to reach acceptable ethanol content was set to a minimum of 10. The process parameters for formulating squalene oil emulsions using the microfluidic chip technology are presented in Figure 1, yielding emulsions (MC160) with the desired physicochemical characteristics. We ensured that formulations had less than 4 EU/mL endotoxin of the adjuvant (corresponding to an acceptable endotoxin limit of 5 EU/kg of mice) [50,51]. These characteristics were found to be comparable to the previously established emulsion (SEA160) (Table 1) and were found to be stable in terms of size, PDI, and squalene content (Figure S2).

### 3.2. Antigen Stability on Mixing with Adjuvants

The stability of the antigen on mixing with the adjuvant is crucial for vaccine efficacy [52,53]. Here, we mimicked the clinical process of mixing the antigen and the adjuvant right before administration and evaluated the stability of the antigen after mixing with the adjuvant [9,10]. For all of our antigen stability work, we used ovalbumin (OVA) as the model antigen as it is water-soluble, can be solubilized in buffers at higher concentrations, and is widely studied for the development and characterization of adjuvant–antigen systems [54,55]. The antigen solution was mixed with the emulsion adjuvant and stored at 4 °C for up to 48 h. Antigen integrity was first evaluated by gel electrophoresis on SDS-PAGE gels. Ovalbumin protein bands were observed at all timepoints (Figure S3). The adjuvant alone was added as a negative control (lane 3) and ovalbumin alone was added as a positive control for the antigen (lane 5). The general smearing observed in the adjuvanted OVA groups (lanes 6–10) was due to the possibility of remnants from the disruption of oil droplets via air-driven ultracentrifugation and could be observed in the emulsion-only lane (lane 5). OVA alone (lane 3; 43 kDa MW) migrated with OVA bands in the emulsions at all timepoints (lanes 6–10), thus suggesting that the protein remained stable in the emulsion-adjuvanted vaccine for 48 h at 4 °C. These data provided sufficient preliminary evidence to establish the integrity of the antigen after mixing with adjuvants.

The structural stability of the antigen when mixed with the adjuvant and storage at 4 °C was determined using far-UV CD measurements (200–260 nm). Visually, all samples mixed with adjuvant overlap with each for both SEA160 and MC160 (Figure S4). Additionally, % NRMSD values were calculated and are shown in Figure S5. These NRMSD values were found to be lower than 10% (acceptance criteria) indicating that there was no change in the structure of the antigen on mixing with the adjuvant. The effect of storage of the adjuvant–antigen mixture on the Tm of OVA was evaluated. The Tm values of these samples using DSF were determined using the first-derivative approach. Compared to OVA alone, no shift in average Tm values on storage with adjuvants was observed (Table S1 and Figure S6). Tm, along with CD and gel electrophoresis, highlighted that the antigen characteristics did not change on mixing with the emulsion adjuvant (MC160 and SEA160) and storage up to 48 h.

### 3.3. In Vivo Immunogenicity of Emulsion Adjuvants Using CMV gB Antigen

**Figure 3:**
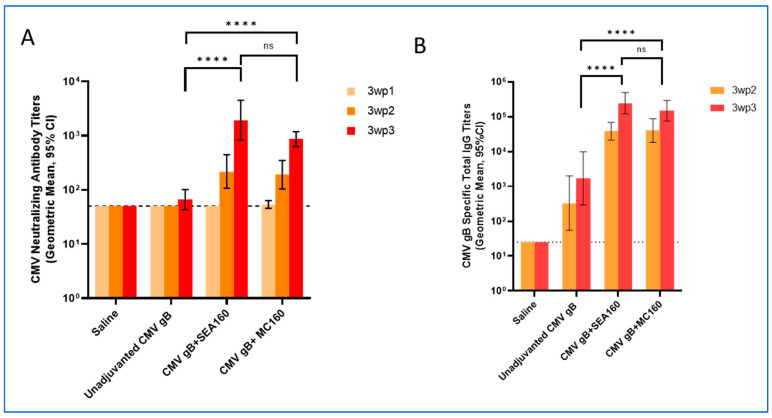
Humoral immune responses in mice immunized with unadjuvanted and adjuvanted CMV gB: (**A**) neutralizing antibody titers; (**B**) CMV-gB-specific total IgG titers. Humoral responses were measured from serum collected at three weeks post 1st (3wp1), three weeks post 2nd (3wp2), and three weeks post 3rd (3wp3) immunization. Each bar represents geometric mean titers (n = 10 mice/group) with a 95% confidence interval (CI). Statistics were performed on titers post-log transformation using one-way ANOVA followed by Tukey’s test to compare groups with each other. The dotted line represents the limit of detection (LOD). Statistical differences marked on the graph are for 3wp3 titers; here, ns = not significant, * = *p* ˂ 0.05, ** = *p* ˂ 0.01, *** = *p* ˂ 0.001, and **** = *p* ˂ 0.0001.

**Figure 4:**
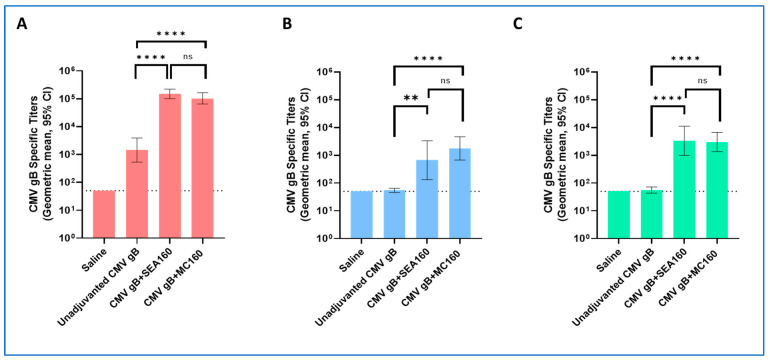
CMV-gB-specific IgG subclass antibody titers ((**A**) IgG1, (**B**) IgG2a, and (**C**) IgG2b) from mice immunized with unadjuvanted and adjuvanted CMV gB. Antibody titers were measured from serum collected three weeks post-third immunization. Each bar represents geometric mean titers (n = 10 mice/group) with a 95% confidence interval (CI). Statistical analyses were performed on titers post-log transformation using one-way ANOVA followed by Tukey’s test to compare all groups with each other. The dotted line represents the limit of detection (LOD). Statistical differences are marked on the graph; here, ns = not significant, * = *p* ˂ 0.05, ** = *p* ˂ 0.01, *** = *p* ˂ 0.001, and **** = *p* ˂ 0.0001.

After formulating, characterizing emulsions, and testing the antigen stability on mixing with the emulsion adjuvant, an in vivo immunogenicity study was performed to compare the adjuvanticity of MC160 to the previously well-established squalene-oil adjuvant SEA160 induced by intramuscular immunization with the CMV gB antigen [21]. CMV gB is a soluble, purified subunit protein with inherently low immunogenicity, making it a suitable candidate for adjuvant screening. Additionally, gB plays a key role in the CMV viral fusion process, infectivity, and cell-to-cell spread, and neutralizing antibodies against gB makes it a suitable candidate for CMV vaccine design [40]. The neutralizing antibody titers were determined in ARPE-19 epithelial cells using the TB-40 CMV virus strain (Figure 3A). A Luminex-based assay was performed to measure anti-CMV gB total IgG (Figure 3B) and subclass IgG (Figure 4) antibodies present in the serum.

Neutralizing antibody titers were considered as the primary readout as neutralizing antibodies are critical in blocking viral entry into the cells [56,57]. CB6F1 mice vaccinated with saline showed no immune response and served as a negative control group. The three weeks following the first immunization titers were overall low for both unadjuvanted as well as adjuvanted groups. Since CMV gB is an antigen with inherently low immunogenicity, these low titers were as expected. Adjuvanted groups (SEA160 and MC160) showed slightly higher titers compared to unadjuvanted CMV gB at three weeks post the second immunization. At three weeks post the third immunization, a considerable increase in titers for adjuvanted groups was observed compared to the unadjuvanted group. Both MC160 and SEA160 showed a similar adjuvant effect, inducing higher mean titers compared to the unadjuvanted group. At three weeks post the second and third immunizations, MC160 and SEA160 showed titers that were not statistically different, highlighting similarity in the adjuvant effect of the two emulsions (Figure 3A).

In addition to the neutralizing antibody titers, CMV-gB-specific total IgG titers in sera were determined for three weeks post the second- and third-immunization timepoints using a Luminex-based assay (Figure 3B). This assay was a secondary readout to evaluate antigen-specific binding antibodies in the sera that are critical for preventing both viral entry as well as cell-to-cell spread [57,58]. The overall trend for groups and timepoints remained similar to the trend observed in neutralizing antibody titers. MC160 showed statistically higher titers compared to the unadjuvanted group. At three weeks post the third immunization, MC160 and SEA160 showed no statistical difference in the titers, highlighting similarity in the immune response generated. Thus, the overall humoral responses (neutralizing antibody responses and binding antibody (total IgG) responses) suggest that MC160 shows an adjuvant effect similar to SEA160, which supports that the process of making emulsions using a microfluidic chip generates effective emulsion adjuvants (Figure 3). To better understand the Th skewing of MC160, IgG subclass titers (IgG1, IgG2a, and IgG2b) against CMV gB were measured in sera at three weeks post the third immunization timepoint using a Luminex-based assay (Figure 4). For all IgG subclasses, unadjuvanted CMV gB showed lower responses compared to the adjuvanted groups. Overall, across total and all IgG subclass titers, MC160 and SEA160 showed comparable responses. MC160, like SEA160, induced a Th2 (high IgG1)-skewed immune response (Figure 4A).

## 4. Discussion

Current manufacturing methods of emulsion adjuvants are complex, high-shear processes and require heavy machinery and maintenance [17,59]. Microfluidic-chip-based technology, which has shown rapid scalability and ease of production especially in terms of COVID-19 vaccines, is a potential alternative approach to manufacturing emulsions with rapid scalability [25,26,27]. At the same time, it can address formulation challenges associated with incorporating other immunomodulatory components into emulsions. The affordability aspects of the microfluidic chip platform make it suitable for deploying technology transfer and simpler and rapid production could improve vaccine access and aid in pandemic preparedness in LMICs. Here, we have demonstrated a potential process for manufacturing nanoemulsions using a microfluidic chip platform. The optimized process yields emulsions (MC160) of sizes ~160 nm with a PDI of less than 0.200. In the past, emulsion-droplet-size-dependent adjuvant impacts have been studied and have shown that emulsions with droplet sizes of 160 nm have shown better adjuvant effects compared to smaller droplet size emulsions (20 and 90 nm) [21,31]. A PDI of less than 0.3 shows the homogenous population of the droplets [60]. Upon storage, we reported that these emulsion droplets were found to be stable for up to four weeks in terms of size and PDI, which are critical as changes in these are indicators of Ostwald ripening [61,62]. Additionally, we observed no significant changes in the squalene content on storage. Ovalbumin was used as a model antigen to further characterize the impact on the antigen upon mixing with the adjuvants [54,55]. Tm, far-UV CD spectra, and gel electrophoresis data revealed that the antigen (ovalbumin) integrity was not compromised on mixing with the adjuvant. This was an important criterion as the adjuvant and antigen are typically mixed right before the administration of the vaccine in a clinical setting [52,53]. The in vivo study was designed to compare the humoral responses induced following the immunization of the CMV gB antigen with MC160, with unadjuvanted CMV gB, and with SEA160. CB6F1 mice were picked to study the nature of immune responses as these are hybrid mice of the C57BL/6 mouse and the Balb/c mouse, and their genetic background with both C57BL/6 and Balb/c strains eliminates the bias in the Th types of immune responses present in the two strains [63]. Neutralizing antibody responses showed a significant increase in the three-weeks-post-third immunization (3wp3) titers for both emulsion groups (MC160 and SEA160) compared to unadjuvanted CMV gB. Similar to neutralizing antibody titers, binding antibody titers against CMV gB (total IgG titers) also showed a significant increase in the titers three weeks post-third immunization (3wp3) for both emulsion groups (MC160 and SEA160). It was observed that MC160 and SEA160 showed equivalent responses at all timepoints for neutralizing antibody titers and antigen-binding antibody titers. CMV-gB-specific IgG subclass titers at 3wp3 serum samples aided in getting deeper insights into the Th skewing responses. A similar trend to binding antibody titers was observed and responses for all three subclasses (IgG1, IgG2a, and IgG2b) were higher for emulsion groups than unadjuvanted groups. IgG1 was the major subclass in the total IgG response, highlighting the Th2 skewed nature of the immune responses, which are critical for antibody-mediated immune responses, for MC160.

## 5. Conclusions

Emulsion adjuvants have well-established safety and have demonstrated potency and efficacy for subunit vaccines [3,5]. Not only do they enhance immune responses and enable antigen dose sparing but they can also be produced rapidly on a large scale to meet an increasing demand. When combined with various antigens, emulsions have proven to be effective adjuvants in humans and have become the preferred choice for influenza vaccines, especially in pandemic situations [32,64]. Our results have established a proof of concept for microfluidic-chip-based technology as an alternative approach to making high-quality emulsion adjuvants for vaccines, which can further accelerate rapid production and deployment for pandemic preparedness, particularly in low-to-middle-income countries, where resources and infrastructure are often limited. MF59 has been used in several clinical and preclinical studies on adjuvanted CMV gB for higher neutralizing and anti-gB IgG titers compared to CMV gB immunized alone [65,66,67]. SEA160 was created using a self-emulsification process with the same goal of simpler manufacturability and availability in LMICs for pre-pandemic and pandemic preparedness [31]. The comparability of MC160 with SEA160, a previously established squalene-only emulsion with similar characteristics and humoral responses, is critical as it underscores the robustness of our approach and ensures that the new method does not compromise the effectiveness of the adjuvant while offering additional benefits. Over two decades of global experience and their crucial role in managing an influenza pandemic, emulsion adjuvants have been established with a solid foundation and represent a benchmark for the development of novel adjuvants for vaccines [1,68]. The potential next steps in our research involve the optimization and validation of the microfluidic chip technology in a large-scale production environment. Industrial partnerships and collaborations with public health organizations will be crucial in bringing this innovation to the market and ensuring integration into existing adjuvant production frameworks. In summary, the development of microfluidic-chip-based technology for emulsion adjuvant production represents an innovation with far-reaching implications. By providing a more efficient, scalable, and cost-effective method, this technology has the potential to transform the landscape of vaccine manufacturing and significantly enhance global access and pandemic preparedness.

## Figures and Tables

**Table 1:** Characterization data set of formulations highlighting particle size, polydispersity index (PDI), endotoxin levels, osmolality, pH, squalene content, and post-TFF ethanol. The data set is presented as means ± std. deviations for three different batches (each measured in technical duplicates).

Parameter	SEA160	MC160
Particle size (nm)	179.8 ± 5.5	156.4 ± 3.4
Polydispersity index (PDI)	0.169 ± 0.028	0.186 ± 0.004
Endotoxin (EU/mL)	<1.00	<1.00
Osmolality (mOsm/kg)	35.5 ± 2.1	50.5 ± 24.8
pH	6.517 ± 0.043	6.557 ± 0.012
Squalene Content (mg/mL)	30.92 ± 0.20	33.11 ± 00.29
Post TFF ethanol (ppm)	-	36.77 ± 1.39

## Data Availability

Data available upon request.

## Supplementary Materials

The following supporting information can be downloaded at: https://www.mdpi.com/article/10.3390/vaccines12121343/s1, Figure S1: Plot of residual ethanol in formulation as a function of diavolumes of buffer exchanged; Figure S2: Short-term stability assessment for microfluidic chip formulation MC160 up to 4 weeks; Figure S3: Gel electrophoresis to assess ovalbumin (OVA) antigen stability on mixing with MC160 and storage at 4 °C up to 48 h; Figure S4: Molar ellipticity plots of ovalbumin with emulsion adjuvant; Figure S5: % NRMSD Values for far UV molar ellipticities; Figure S6: Tm of antigen-adjuvant mixture determined from DSF; Table S1: Tm of antigen-adjuvant mixture determined from DSF.
