# Supplementary material for: Microfluidic-Chip-Based Formulation and In Vivo Evaluations of Squalene Oil Emulsion Adjuvants for Subunit Vaccines
Source: Vaccines (Basel). 2024 Nov 28;12(12):1343. doi: 10.3390/vaccines12121343 (PMC11680198; doi:10.3390/vaccines12121343)
Supplement: Supplementary file 1 [file vaccines-12-01343-s001.zip › vaccines-3257411-supplementary.pdf]

vaccines

MDPI

SUPPLEMENTAL INFORMATION

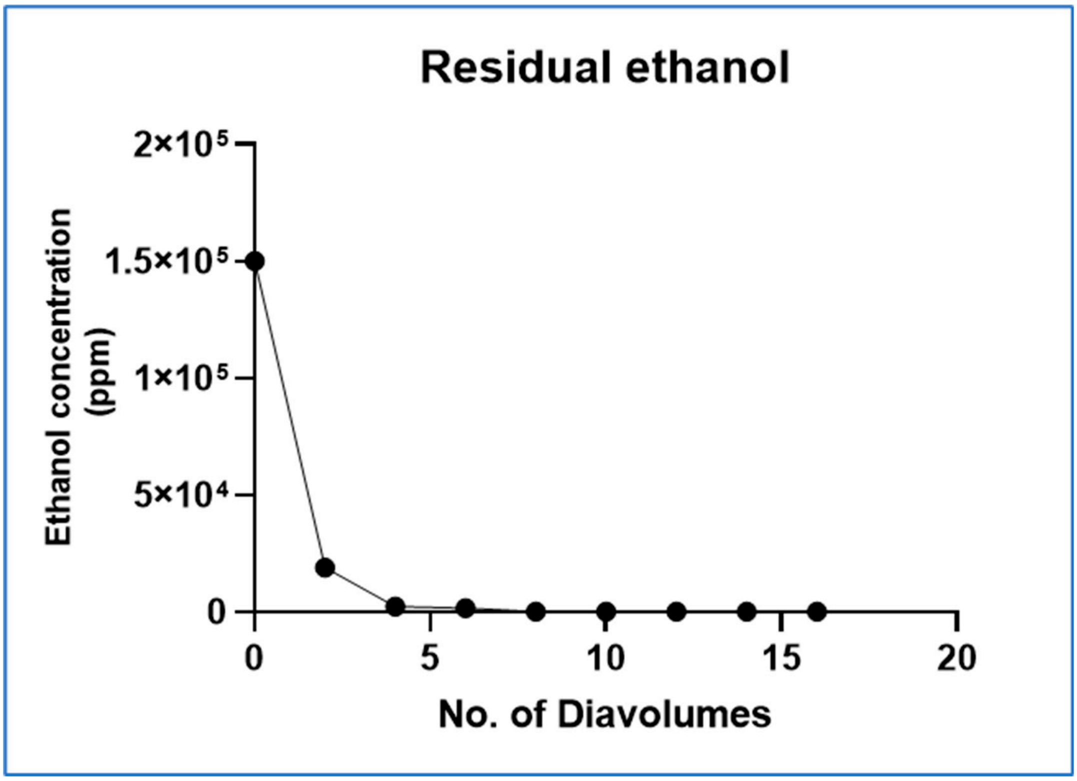

Figure S1. Plot of residual ethanol in formulation as a function of diavolumes of buffer exchanged. The data is presented as mean (± SD) of technical duplicates.

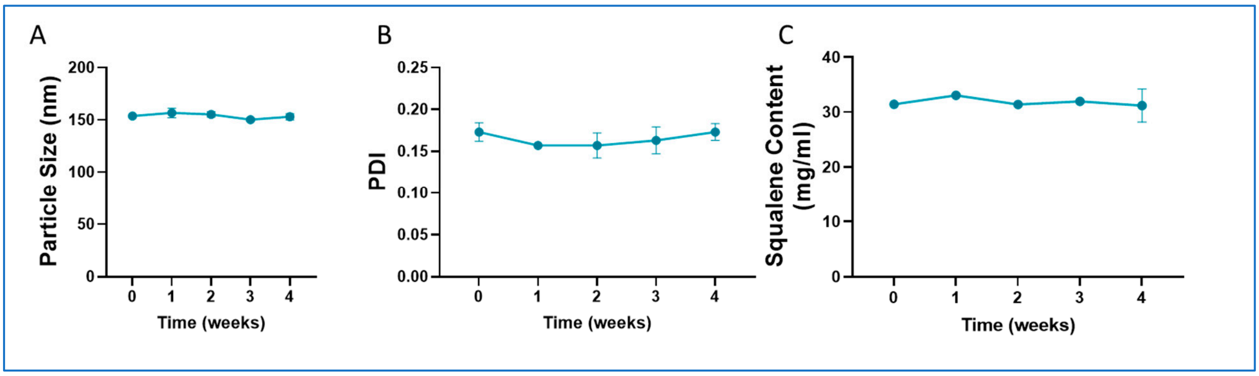

Figure S2. Short term stability assessment for microfluidic chip formulation MC160 up to 4 weeks. The data are presented as mean ± SD. Particle size and polydispersity index (PDI) are presented as technical triplicates, and squalene content is presented as technical duplicates.

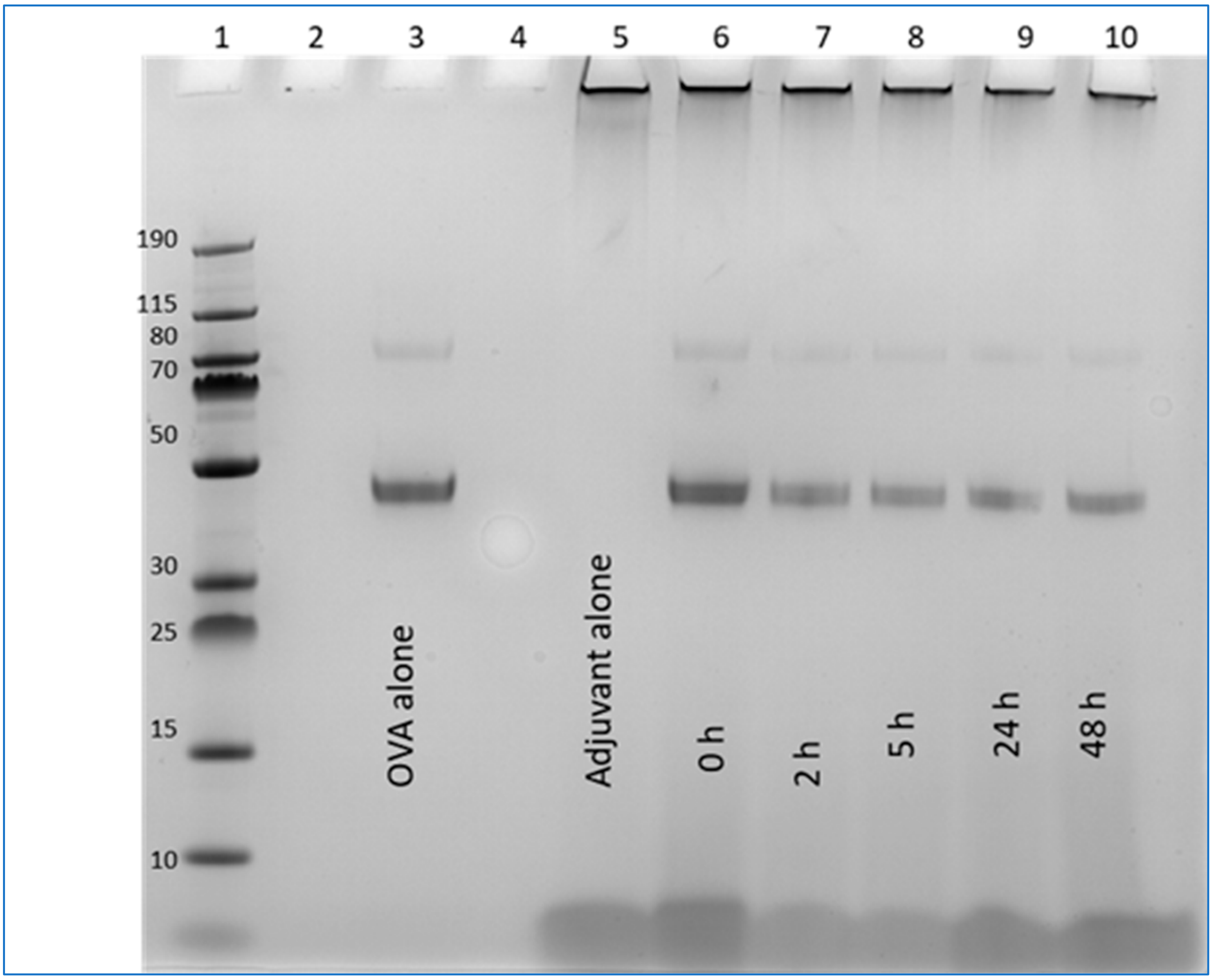

Figure S3. Gel electrophoresis to assess ovalbumin (OVA) antigen stability on mixing with MC160 and storage at 4 ⁰C up to 48 hours.

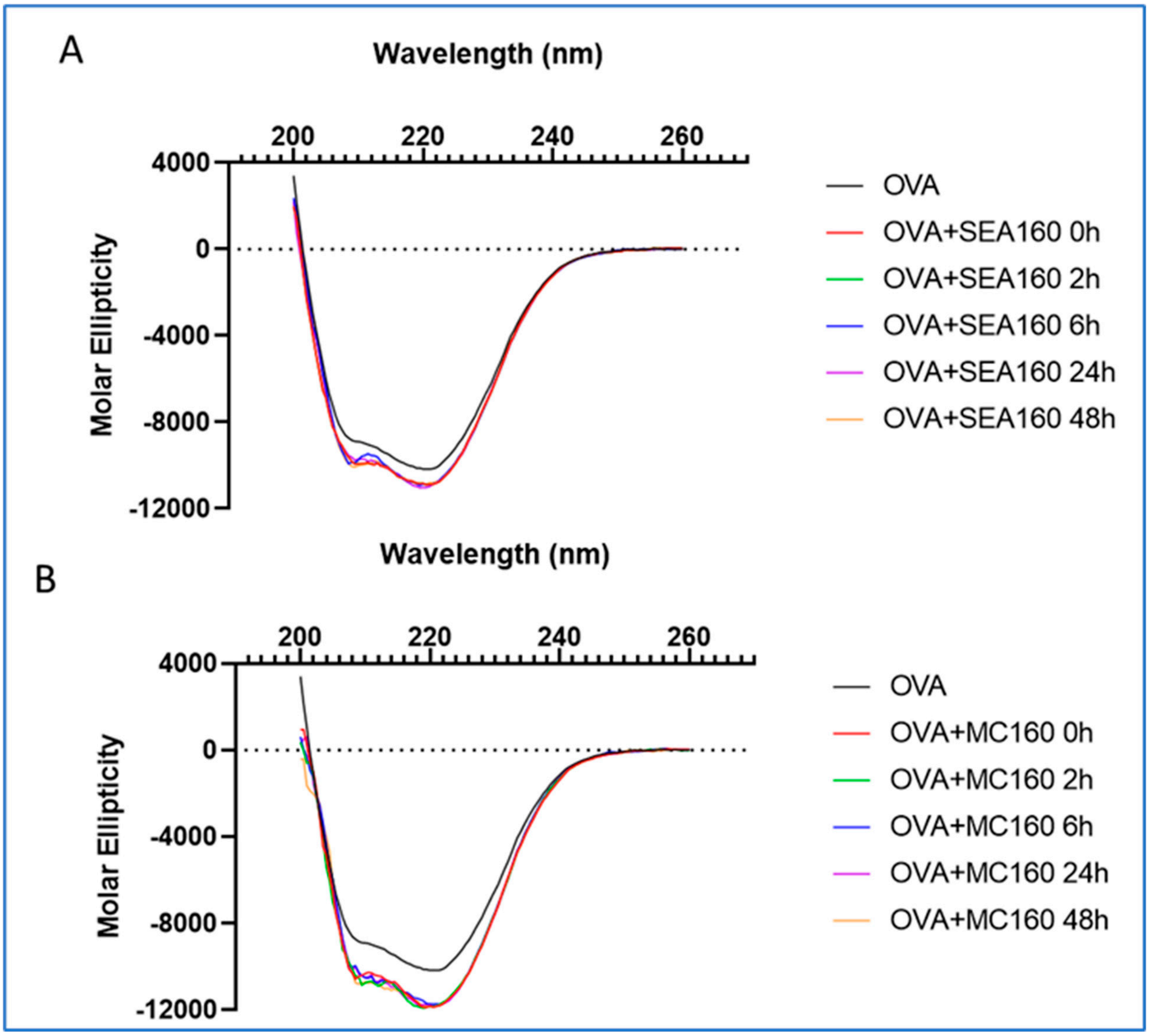

Figure S4. Molar ellipticity plots of Ovalbumin emulsion adjuvant. Panel A: SEA160 samples with OVA, Panel B: MC160 samples with OVA. The data set is presented as the mean for two accumulations of three cycles for each sample.

A

Sample name	OVA+SEA160 0h	OVA+SEA160 2h	OVA+SEA160 6h	OVA+SEA160 24h	OVA+SEA160 48h
OVA+SEA160 0h	2.129				
OVA+SEA160 2h	0.021	2.277			
OVA+SEA160 6h	1.744	1.737	2.280		
OVA+SEA160 24h	1.307	1.304	1.282	1.696	
OVA+SEA160 48h	1.432	1.429	0.280	1.195	2.656

B

Sample name	OVA+MC160 0h	OVA+MC160 2h	OVA+MC160 6h	OVA+MC160 24h	OVA+MC160 48h
OVA+MC160 0h	2.74				
OVA+ MC160 2h	1.560	4.106			
OVA+ MC160 6h	1.739	1.905	4.427		
OVA+MC160 24h	1.322	1.526	1.449	3.044	
OVA+MC160 48h	2.857	2.517	2.019	2.738	3.623

Figure S5. % NRMSD Values for far UV molar ellipticities. Panel A: SEA160 samples with OVA, Panel B: MC160 samples with OVA The columns represent samples and rows represent references used for calculations.

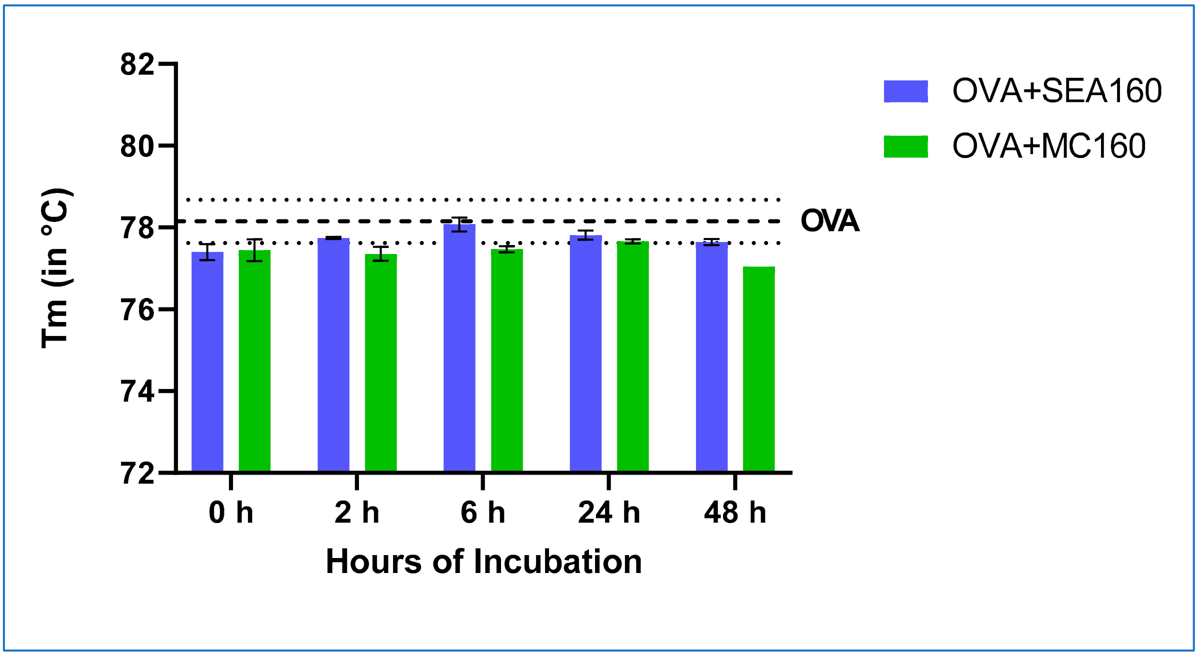

Figure S6. Tm of antigen-adjuvant mixture determined from DSF. The data are presented as mean ± SD of technical duplicates.

Sample Name	Tm (°C)
Ovalbumin	78.15 ± 0.53 °C
Ovalbumin + SEA160 0h	77.40 ± 0.20 °C
Ovalbumin + SEA160 2h	77.75 ± 0.03 °C
Ovalbumin + SEA160 6h	78.08 ± 0.17 °C
Ovalbumin + SEA160 24h	77.82 ± 0.17 °C
Ovalbumin + SEA160 48h	77.65 ± 0.07 °C
Ovalbumin + MC160 0h	77.44 ± 0.27 °C
Ovalbumin + MC160 2h	77.36 ± 0.17 °C
Ovalbumin + MC160 6h	77.47 ± 0.07 °C
Ovalbumin + MC160 24h	77.66 ± 0.05 °C
Ovalbumin + MC160 48h	77.05 ± 0.001°C

Table S1. Tm of antigen-adjuvant mixture determined from DSF. The data are presented as mean ± SD of technical duplicates.
